# “But the moment they find out that you are MSM…”: a qualitative investigation of HIV prevention experiences among men who have sex with men (MSM) in Ghana’s health care system

**DOI:** 10.1186/s12889-017-4799-1

**Published:** 2017-10-03

**Authors:** Sameer Kushwaha, Yasmin Lalani, Geoffrey Maina, Adedotun Ogunbajo, Leo Wilton, Thomas Agyarko-Poku, Yaw Adu-Sarkodie, Francis Boakye, Nanhua Zhang, LaRon E. Nelson

**Affiliations:** 1 University of Toronto, Toronto, ON Canada; 2 University of Rochester, Rochester, NY USA; 3 University of Saskatchewan, College of Nursing, 214-1301 Central Avenue, Prince Albert, SK Canada; 4 Brown University, Providence, RI USA; 5 State University of New York at Binghamton, Binghamton, NY USA; 6 University of Johannesburg, Johannesburg, South Africa; 7 Kwame Nkrumah University of Science & Technology, Kumasi, Ashanti Ghana; 8 Priorities on Rights and Sexual Health, Accra, Ghana; 9 Division of Biostatistics & Epidemiology, Cincinnati Children’s Hospital Medical Center, Cincinnati, OH USA; 10 St. Michael’s Hospital, Centre for Urban Health Solutions, Toronto, ON Canada

**Keywords:** Ghana, HIV prevention, Health care providers, Men who have sex with men (MSM), Stigma, Self-determination theory, Health care climate, Sexual health

## Abstract

**Background:**

The prevalence of HIV in Ghana is 1.3%, compared to 17% among men who have sex with men (MSM). There is limited empirical data on the current health care climate and its impact on HIV prevention services for Ghanaian MSM. The purposes of this study were to investigate (1) MSM’s experiences using HIV prevention resources, (2) what factors, including health care climate factors, influenced MSM’s use of prevention resources and (3) MSM self-identified strategies for improving HIV/sexually transmitted infection (STI) prevention among MSM in Ghanaian communities.

**Methods:**

We conducted 22 focus groups (*n* = 137) with peer social networks of MSM drawn from three geographic communities in Ghana (Accra, Kumasi, Manya Krobo). The data were examined using qualitative content analysis. Interviews with individual health care providers were also conducted to supplement the analysis of focus group findings to provide more nuanced illuminations of the experiences reported by MSM.

**Results:**

There were four major findings related to MSM experiences using HIV prevention resources: (1) condom quality is low, condom access is poor, and condom use is disruptive, (2) inaccurate information undermines HIV testing (3), stigma undermines HIV testing, and (4) positive attitudes towards HIV prevention exist among MSM. The main healthcare climate factors that affected prevention were that MSM were not free to be themselves, MSM were not understood by healthcare providers, and that MSM did not feel that healthcare providers cared about them. To improve HIV prevention MSM suggested increased education tailored to MSM should be provided to enable self-advocacy and that education and awareness are needed to protect human rights of MSM in Ghana.

**Conclusion:**

MSM in Ghana are exposed to negative health care climates. Health care spaces that are unsupportive of MSM’s autonomy undermine the uptake of prevention measures such as condoms, HIV testing, and accurate sexual health education. These findings contribute to knowledge to inform development of HIV prevention interventions for MSM in Ghana, such as culturally appropriate sexual health education, and digital technology to connect individuals with resources supportive of MSM.

## Background

Despite considerable efforts to end the human immunodeficiency virus (HIV) pandemic, many countries struggle to achieve the United Nation’s Millennium Development Goals to reduce the number of new HIV infections and mortality from acquired immune deficiency syndrome (AIDS) [1]. This is particularly evident among key populations who bear a disproportionate burden of the global HIV prevalence, such as men who have sex with men (MSM). In Ghana, it is estimated that 1.3% of the general adult population lives with HIV [2, 3]. This is in contrast to a prevalence of 17.5% among Ghanaian MSM, [4, 5] which is nearly 15 times higher than in the general population. There are additional regional disparities in HIV prevalence among MSM with estimates as high as 34.3% in the Greater Accra region and 13.7% in the Ashanti metropolitan region [4].

Prior research has indicated that a constellation of factors influence HIV-related health disparities for Ghanaian MSM [4–6]. A 2012 nationwide biobehavioral surveillance study found that less than 50% of MSM in Ghana had any exposure to HIV prevention programming and services [4]. Another study examining HIV risk factors of MSM in Ghana found that a high proportion of men in their sample (93%) had more than one partner (*M* = 5.11, *SD* = 7.4) in the past six months prior to assessment and reported low levels of knowledge regarding the transmission and prevention of HIV and other sexually transmitted infections (STIs) [6]. Additionally, there is increasing evidence that the use of sex as a commodity in exchange for financial resources is a prevalent practice, especially among young MSM – a pattern that is consistent in MSM populations across sub-Saharan Africa [7–9]. This is concerning in light of evidence that MSM engaging in transactional sex are at greater risk of HIV infection [10]. All of these dynamics, combined with the high concentration of HIV in Ghana’s MSM population, explain some of the factors that have perpetuated HIV disparities among MSM.

Prevailing social and legal structural barriers affecting HIV-infected individuals and MSM, such as criminalization, social isolation [11], and financial exclusion [11, 12], intersect to complicate HIV prevention efforts for MSM in Ghana [13] and elsewhere in West Africa [7]. First, it is unclear and debated whether same-gender sexual practices of MSM are afforded basic protection from discrimination and persecution under Ghana’s legal system [14]. Although the Ghanaian *Criminal Code* does not explicitly prohibit homosexuality, Section 104 of the *Code* states “Unnatural carnal knowledge is sexual intercourse with a person in an unnatural manner…”. The ambiguity in law, according to prominent non-governmental organizations (NGOs) (e.g., Gay and Lesbian Association of Ghana [GALAG] and the Center for Popular Education and Human Rights Ghana [CPEHRG]) has been used to justify the imprisonment of men who engage in male-to-male sexual behavior [15]. Furthermore, this reflects long-standing narratives that broad societal attitudes in Ghana are generally hostile to same-gender sexualities, behavioral practices, relationships, and communities [16, 17]. These narratives, combined with HIV-related stigma, contribute to a social context that perpetuates structural barriers that marginalize MSM. There are various forms of stigma that MSM experience in both their public and private lives that can undermine HIV prevention efforts [6]. HIV seronegative MSM can experience HIV stigma vicariously through an intersecting belief that their community attributes shame, disgust, and dishonor to HIV-infected individuals, and that MSM will eventually be (and deserve to be) infected with HIV as a divine punishment for perceived sins [6].

The public health care system in Ghana is the cornerstone of providing comprehensive HIV prevention education and clinical prevention services to MSM and other key populations at disproportionate risk for HIV infection [18]. However, the health care system is situated within and influenced by the broader social contexts, wherein health care personnel may possess stigmatizing attitudes towards MSM; these individuals may also be stigmatized for participating in the care of MSM and HIV-infected patients [19]. While there is a growing evidence-base from epidemiologic and behavioral surveillance data regarding behavioral risks and HIV-knowledge in MSM, there is still a critical need for qualitative studies that investigate how factors related to the health care system facilitate or impede HIV prevention for MSM in the Ghanaian context. The purposes of this study were to investigate: (1) MSM’s experiences using HIV prevention resources, (2) what factors, including health care climate factors, influenced MSM’s use of prevention resources, and (3) MSM self-identified strategies for improving HIV prevention among MSM in Ghanaian communities. For this study, two sources of qualitative data were used to understand the aforementioned domains from three key regions in Ghana. Our primary data source was from focus groups with MSM and supplemented by data from individual interviews with health care providers.

## Methods

### Study design

We conducted an interpretive description study using qualitative focus group data from the Kumasi & Accra Project to Prevent AIDS (KAPPA) — an embedded multiple case study conducted between March and June 2012 in three Ghanaian communities: Accra, Kumasi, and Manya Krobo. This study analyzes a subset of the larger KAPPA dataset. Interpretive description is an approach to qualitative study in which the investigator/analyst leverages their clinical training and practice to understand the interplay of a constellation of socio-cultural, psychosocial, and biological factors in how people experience health and illness [20, 21]. A community-based participatory approach was also employed, utilizing input from community researchers, research assistants, and stakeholders with expertise in MSM and HIV prevention in Ghana to inform all parts of the study design, implementation, and analysis. Using interpretive description, combined with community-based participatory approaches, we sought to investigate the research questions in a way that would be most likely to yield findings that could be readily utilized to guide future preventative health and clinical practices related to HIV. The study protocol was approved by the Kwame Nkrumah University of Science and Technology Committee on Human Research, Publication, and Ethics and the University of Toronto HIV Research Ethics Board.

### Recruitment and consent

Local NGO outreach workers and popular opinion leaders who self-identified as MSM carried out recruitment using a chain-referral (or “snowball”) sampling method to recruit Ghanaian MSM. Chain-referral was chosen due to the sensitive nature of the research topic and the difficulty of reaching MSM due to the highly stigmatized nature of same-gender sexual activity in Ghana [22, 23]. This process involved recruiting MSM individuals who were known to NGO outreach workers and opinion leaders. The individual recruits were then asked to invite a group of MSM peers from their social network to participate in a focus group discussion. Recruiters screened potential participants to ensure that they were indeed MSM and not feigning eligibility for an ulterior purpose (e.g. financial incentives). The purpose and expectations of involvement in the study were explained to prospective participants in the language with which they were most comfortable communicating (e.g. English, Twi, Ga, Hausa). Informed consent was then documented by having participants provide a digital signature directly onto a secure platform on an iPad.

Inclusion criteria at the level of the peer-networks included: confidence among the NGO outreach workers that the network was comprised of peers, confidence that the involved men were MSM, and that the network comprised at least four willing study participants. At the time of enrollment, MSM participants were 18 years or older, Ghanaian citizens, biologically male at birth, currently self-identified as cisgender men, and reported sexual activity with another man at least once within the previous six months. All participants received a one-time incentive of 25 New Ghana Cedis (roughly 14 US Dollars) for enrollment, regardless of whether they completed the study [24].

In an effort to further contextualize the healthcare-related experiences of Ghanaian MSM and as a basis to illuminate nuanced understandings of their reports, we conducted individual interviews with Ghanaian healthcare providers that were identified by the men in our study. For example, members of the MSM focus groups identified health care providers whom they had interacted with in the past, regardless of the provider’s attitudes towards MSM. These health care providers were then contacted by local NGO outreach workers and invited to participate in individual interviews. Prior to interviewing health care providers, the process of explaining the study purpose, expectations of their involvement, and obtaining informed consent were the same as for MSM focus group participants.

### Data collection

First, based on a semi-structured focus group guide, data were collected through focus group discussions with MSM; probes were used to help frame the discussion. Ghanaian facilitators were given prior training on qualitative data collection and led all focus group discussions. Focus groups explored topics including: community norms and stigma (related to sexual orientation and HIV), psychosocial and tangible resources within their social networks, and health care climate experiences. Sample question prompts included “How would you describe the community in which you live?” and “Do you think that the health care provider cares about what happens to you? Why?” Additional sample questions asked to MSM focus groups within each topic domain are presented in Table 1.

**Table 1:** Discussion topics, questions, and sample prompts from MSM focus groups

Topic domain	Question	Sample prompts
Community Norms and Stigma	How would you describe the community in which you live?	• What have been some of the obstacles or challenges that you’ve experienced in your community as MSM?
Network Mental Resources – Health Seeking Attitudes (STI/HIV)	What are your thoughts about getting tested for STIs?	• Have you ever been tested [for HIV]? • What prompts you to get tested [for HIV]?
Network Mental Resources – Condom Use Attitudes	Tell us about how often you use condoms now during sex?	• How do you feel about using condoms during sex? o With men? o With women?
Network Mental Resources – Gender Equitable Norms	What are your thoughts about women who carry condoms?	How would you feel if your female partner asked you to use a condom?
Network Mental Resources – Competence Support	What are the HIV/STI prevention needs of guys like you and your friends?	• What are the needs you think you have within your social network?
Network Tangible Resources	What are other needs you have in your life?	• How do you earn money?
Health Care Climate Experiences – Autonomy Support	Who do you go see when you have a sexual health related issue?	• What do you like/not like about the provider that you visit? • Do you feel like you have a say in what happens to you there? • How would you like the experience to be different?
Health Care Climate Experiences – Relatedness Support	Do you think that the health care provider cares about what happens to you?	• Why do you think so? • Why don’t you think so? • Do you want to do what they advise? Why? Why not?
Health Care Climate Experiences – Competence Support	When you leave the health office how capable do you feel to follow provider’s instructions?	• Do you want to do what they advise? Why? Why not?

Following this process, individual interviews were conducted with healthcare providers based on a conversational technique that elicited their perspectives on the HIV prevention needs of Ghanaian MSM. Facilitators for these interviews were also Ghanaians who received training on qualitative data collection. Sample questions included “How does your hospital receive patients with different sexualities?” and “What are some of the HIV prevention needs of men who have sex with men?” A detailed description of the data source for the health care provider transcripts is reported elsewhere [25].

All focus groups and healthcare provider interviews were digital audio-recorded and transcribed verbatim by professional multilingual transcriptionists in Ghana. Transcriptionists were briefed on the sensitive nature of the data and required to sign confidentiality agreements prior to being granted access to audio data. Data were transcribed using Transcriva (V. 2.016). Local facilitators simultaneously translated the transcripts to English, whenever necessary.

### Data analysis

We conducted qualitative content analysis on data generated from focus groups with MSM and healthcare provider interviews. Qualitative content analysis entails a procedural approach in which data is categorized and reviewed iteratively to develop conclusions based on both explicit and implicit meaning in the text [26]. The authors used NVivo for Mac (V. 10.2.1) to manage the qualitative data. Two study authors (SK and LEN) read and reviewed all study transcripts and independently coded data into different NVivo nodes. Data were coded using “open coding”, a process in which additional codes were made as needed throughout the process of reviewing transcripts. After all focus groups and individual interviews were coded, relevant quotes were further refined and organized into broad categories (e.g. “MSM-identified barriers to HIV prevention”) using a spreadsheet created in Microsoft Excel. The spreadsheet facilitated analysis of code clusters within the qualitative data. Using the resulting spreadsheet, the study authors explored the data from to develop central findings.

The excerpts from the individual interviews with health care providers were incorporated solely as an adjunctive strategy to the presentation of focus group findings. The integration of health care provider quotes supports the contextualization of the findings rather than an attempt to compete with MSM as the primary voices represented in the findings. The selective inclusion of health care provider quotes were guided by dyadic analysis techniques in which we examined the focus groups findings and health care provider interview transcripts for similarities and contrasts in their texts and subtexts [27]. After incorporating the excerpts, findings were discussed among all co-authors to ensure a balanced and accurate representation of the data from focus groups.

### Addressing positionality

Many steps were taken to ensure that the findings presented in this paper are accurate and relevant to the cultural contexts of Ghanaian MSM. Positionality refers to the unique identities, experiences, and biases that researchers bring to the design, implementation, and analysis of a given study [28]. The KAPPA study was designed and implemented in partnership with Ghanaians, many of whom identified as MSM, from community based organizations and NGOs. Furthermore, all focus group facilitators were Ghanaian MSM who were able to converse in local languages and knowledgeable about customs and traditional practices. This paper’s authors also represent a diverse group of analysts, including several Ghanaians who work closely with MSM. All authors contributed to the analytic process. SK, YL, GM, and LEN were the primary analysts. Throughout the analytic process, preliminary findings were discussed with staff at the Centre for Popular Education & Human Rights Ghana (CEPEHRG) and Priorities and Rights in Sexual Health (PORSH)—the two largest and longest running LGBT health and human rights organizations in Ghana. Additionally, multiple authors (SK, YL, LEN, GM) reviewed the transcripts and met regularly to discuss and refine the content analysis.

## Findings

The qualitative dataset included transcripts from twenty-two focus groups conducted across the communities of Accra (8 focus groups; *n* = 53), Kumasi (8 focus groups; *n* = 51), and Manya Krobo (6 focus groups; *n* = 33). Most (69%) participants were younger than 25 years of age. Two-thirds (66%) of the men in the study sample had attained an education at or above the 12th grade level. Three-quarters (75%) self-reported that they were HIV negative, although only 69% reported ever being tested for HIV in their lifetime. One-quarter (24%) opted not to report their HIV status. Only one participant self-reported having an HIV infection. Additional detailed demographic data for MSM study participants are reported in Table 2 and elsewhere [6].

**Table 2:** Demographic features of each MSM focus group in Accra, Kumasi, and Manya Krobo

	Accra									Kumasi									Manya Krobo							
Group No.	1	2	3	4	5	6	7	8	Total (Accra)	1	2	3	4	5	6	7	8	Total (Kumasi)	1	2	3	4	5	6	Total (Manya Krobo)	Total (all sites)
Sample Size (n=)	8	6	6	7	6	6	6	8	53	6	8	5	6	6	6	8	6	51	4	6	6	7	6	4	33	137
Age (years)																										
25 or less	*88*	*67*	*17*	*71*	*33*	*50*	*33*	*100*	*60*	*17*	*100*	*80*	*100*	*100*	*83*	*100*	*100*	*86*	*25*	*83*	*83*	*86*	*17*	*–*	*55*	*69*
26–35	*13*	*17*	*50*	*14*	*67*	*50*	*67*	*–*	*32*	*83*	*–*	*20*	*–*	*–*	*17*	*–*	*–*	*14*	*75*	*17*	*17*	*14*	*83*	*100*	*45*	*28*
> 35	*–*	*17*	*33*	*14*	*–*	*–*	*–*	*–*	*8*	*–*	*–*	*–*	*–*	*–*	*–*	*–*	*–*	*–*	*–*	*–*	*–*	*–*	*–*	*–*	*–*	*3*
Education Level																										
< Primary 10th	*13*	*–*	*–*	*–*	*50*	*17*	*67*	*13*	*19*	*–*	*–*	*–*	*–*	*–*	*–*	*–*	*–*	*–*	*75*	*83*	*33*	*43*	*67*	*25*	*55*	*20*
Primary 10th	*38*	*17*	*33*	*29*	*33*	*50*	*17*	*25*	*30*	*–*	*–*	*–*	*17*	*–*	*–*	*–*	*–*	*2*	*–*	*–*	*–*	*14*	*–*	*–*	*3*	*13*
Diploma	*50*	*83*	*50*	*71*	*17*	*33*	*17*	*63*	*49*	*33*	*75*	*–*	*83*	*–*	*33*	*25*	*67*	*41*	*–*	*–*	*67*	*43*	*–*	*–*	*21*	*39*
College	*–*	*–*	*17*	*–*	*–*	*–*	*–*	*–*	*2*	*67*	*25*	*100*	*–*	*100*	*67*	*75*	*33*	*57*	*25*	*17*	*–*	*–*	*33*	*75*	*21*	*27*
HIV Serostatus																										
HIV negative	*63*	*67*	*100*	*86*	*100*	*100*	*100*	*100*	*89*	*33*	*25*	*40*	*83*	*33*	*33*	*75*	*67*	*49*	*75*	*83*	*100*	*100*	*83*	*100*	*91*	*74*
HIV positive	*–*	*17*	*–*	*–*	*–*	*–*	*–*	*–*	*2*	*–*	*–*	*–*	*–*	*–*	*–*	*–*	*–*	*–*	*–*	*–*	*–*	*–*	*–*	*–*	*–*	*1*
Status unknown	*25*	*–*	*–*	*14*	*–*	*–*	*–*	*–*	*6*	*50*	*75*	*60*	*17*	*67*	*67*	*25*	*33*	*49*	*25*	*17*	*–*	*–*	*17*	*–*	*9*	*23*
Decline to answer	*13*	*17*	*–*	*–*	*–*	*–*	*–*	*–*	*4*	*17*	*–*	*–*	*–*	*–*	*–*	*–*	*–*	*2*	*–*	*–*	*–*	*–*	*–*	*–*	*–*	*2*
HIV Testing History																										
Never Tested	*38*	*–*	*–*	*14*	*–*	*–*	*17*	*–*	*9*	*67*	*75*	*80*	*83*	*50*	*83*	*50*	*50*	*67*	*–*	*17*	*–*	*–*	*33*	*25*	*12*	*31*
Tested ≥1 time	*63*	*83*	*100*	*86*	*100*	*100*	*83*	*100*	*89*	*33*	*25*	*20*	*17*	*50*	*17*	*50*	*50*	*33*	*100*	*83*	*100*	*100*	*67*	*75*	*88*	*68*
Sexual Attraction																										
Men only	*25*	*33*	*17*	*43*	*67*	*50*	*100*	*100*	*55*	*83*	*75*	*100*	*67*	*67*	*67*	*88*	*100*	*80*	*25*	*–*	*17*	*71*	*17*	*–*	*24*	*57*
Men and Women	*75*	*67*	*67*	*57*	*33*	*50*	*–*	*–*	*43*	*17*	*25*	*–*	*33*	*33*	*33*	*13*	*–*	*20*	*75*	*100*	*83*	*29*	*83*	*100*	*76*	*42*
Women only	*–*	*–*	*17*	*–*	*–*	*–*	*–*	*–*	*2*	*–*	*–*	*–*	*–*	*–*	*–*	*–*	*–*	*–*	*–*	*–*	*–*	*–*	*–*	*–*	*–*	*1*
Housing																										
Renting	*50*	*17*	*33*	*14*	*67*	*17*	*67*	*–*	*32*	*50*	*13*	*60*	*–*	*–*	*17*	*–*	*–*	*16*	*75*	*50*	*50*	*29*	*33*	*25*	*42*	*28*
Live with parents	*38*	*67*	*67*	*71*	*33*	*50*	*33*	*100*	*58*	*50*	*63*	*40*	*100*	*17*	*33*	*25*	*–*	*41*	*25*	*50*	*50*	*43*	*33*	*25*	*39*	*47*
Boarding House	*–*	*–*	*–*	*–*	*–*	*–*	*–*	*–*	*–*	*–*	*25*	*–*	*–*	*83*	*50*	*75*	*100*	*43*	*–*	*–*	*–*	*29*	*–*	*–*	*6*	*18*
Squatting	*–*	*–*	*–*	*–*	*–*	*17*	*–*	*–*	*2*	*–*	*–*	*–*	*–*	*–*	*–*	*–*	*–*	*–*	*–*	*–*	*–*	*–*	*–*	*–*	*–*	*1*
Home Owner	*13*	*17*	*–*	*14*	*–*	*17*	*–*	*–*	*8*	*–*	*–*	*–*	*–*	*–*	*–*	*–*	*–*	*–*	*–*	*–*	*–*	*–*	*33*	*50*	*12*	*6*
Marital Status																										
Not Married	*100*	*100*	*83*	*100*	*100*	*100*	*100*	*100*	*98*	*100*	*100*	*100*	*100*	*100*	*100*	*100*	*100*	*100*	*100*	*100*	*100*	*100*	*50*	*75*	*88*	*96*
Married	*–*	*–*	*17*	*–*	*–*	*–*	*–*	*–*	*2*	*–*	*–*	*–*	*–*	*–*	*–*	*–*	*–*	*–*	*–*	*–*	*–*	*–*	*50*	*25*	*12*	*4*

Numbers in italics are expressed as percentages

Various types of health care providers (*n* = 25) were interviewed in the three cities, including nurses, physicians, and counselors. Demographic features of the health care providers are further detailed in Table 3.

**Table 3:** Demographic data for interviewed health care providers

Characteristics	Occupation								
	A	B	C^a^	D	E^b^	F	G	H	I
Gender									
Man		•	••		••	•	•	•	•••••
Woman	•		••	•	••••••				••
Community									
Accra	•			•	•••••••			•	
Kumasi		•	•••		•	•	•		•••
Manya Krobo			•						••••

A = Counselors, B = hospital administrator, C = lab technician/scientist, D = midwife, E = Nurse, F = pharmacist, G = physician, H = physician assistant, I = Health care worker, not otherwise specified. Note: Each dot (•) symbol represents one person

^a^Includes biomedical scientists, medical lab technicians, and medical statisticians

^b^Includes different specializations (e.g. public health, sexual health), as well as nurses with training in counseling and/or midwifery

### MSM experiences using HIV prevention resources

Low access to and negative attitudes towards HIV risk-reduction strategies and testing hindered HIV prevention. MSM expressed disdain towards basic HIV prevention tools - namely condoms, lubricants, and HIV testing – due to their poor quality, inaccessibility, and lack of confidence in their efficacy as HIV prevention tools. As shown in Table 4, many of the focus groups also discussed positive views towards HIV prevention tools, although negative sentiments were typically more dominant throughout the groups.

**Table 4:** Focus group discussions contributing data to findings from Accra, Kumasi, and Manya Krobo, Ghana

Findings and Supporting Data	Accra (*n* = 8)^a^	Kumasi (n = 8)^a^	Manya Krobo (*n* = 6)^a^
1. MSM Experiences Using HIV Prevention Resources			
Condom Quality is Low, Condom Access is Poor and Condom Use is Disruptive	•••••••	•••••••	••••
Stigma and Inaccurate Information Undermine HIV Testing	•	•••••••	••
Positive Attitudes Towards HIV Prevention Exist Among MSM	•••••••	•••	••••
2. Health Care Climate Factors that Influence Use of HIV Prevention			
MSM Are Not Free to Be Themselves	•••	•••••	•
MSM Are Not Understood By Health Care Providers	••	••••••	•
MSM Do Not Feel that Health Care Providers Care About Them	••	•••••••	•
3. Strategies for Improving HIV/STI Prevention among MSM			
HIV Prevention Education Tailored to MSM Enables Self-advocacy	••	•	•
Education and Awareness to Protect Human Rights of LGBT Ghanaians	•••••	•••••	

Each dot represents one focus group that contained data supporting the particular finding

Dots are a measure of frequency, but not necessarily of the amount of content within a particular focus group supporting the finding

^a^Refers to number of focus groups, not individual participants

#### Condom quality is low, condom access is poor and condom use is intermittent

A major reason for low condom use identified across the groups was that condoms detracted from the pleasure of sex, either by decreasing sensation or because it feels “unnatural”. The process of finding and putting on a condom was also identified as disengaging from the sexual experience. In the excerpt below, a participant explains how, despite understanding their importance for HIV prevention, the inconvenience and discomfort of condoms deters him from using them:

**MSM Respondent:** [Condoms are] necessary to prevent STDs [sexually transmitted diseases] but I don’t think I’ve used it before… It’s a question of convenience and comfort. It’s not comfortable.

**Facilitator:** Would you use it if you were sleeping with a woman?

**MSM Respondent:** If I were sleeping with a woman? I don’t think I will still feel comfortable… As I said, there should be a direct contact. Nothing should be an intermediate. That is the only way you can make full satisfaction.

-Kumasi, Focus Group 5

Higher quality condoms, which were perceived to be more protective and pleasurable, were described as expensive, in short supply, and out of reach to many MSM. They were also dissuaded from using more readily available “low quality” condoms because they doubted their effectiveness at reducing the risk of acquiring HIV. Some participants held these beliefs based on personal experiences with condoms breaking during sex, while others heard of negative experiences from peers.

Concerns about poor quality condoms were corroborated by health care providers who noted that condom accessibility and poor quality were frequent concerns from their MSM patients. Below, a health care provider discussed how after their clinic ended a free lubricant and condom program several patients were unable to continue using them due to their prohibitive cost. He explains that health care professionals are limited further by the fact that although they have condoms to sell, they do not have lubricants to offer patients.

**Health Care Provider (HCP) (Senior Nursing Officer):** Some time back, when we were on a project, they gave us the lubes and the condoms, which we [gave] to them, free of charge. When that project ended, now the condoms that we are getting, it’s not for free. Then we sell them. Some of them cannot afford. [Also], we don't have the lubes and therefore we talk about the use of condoms and the lubes but you cannot provide to the clients because of limitations. We don't have many. What we have, we sell and some of those folks don't have money.

-Accra, Health Care Provider Interview 9

Trust was another factor that influenced participants’ decisions to use condoms. Participants reported the longer the time in a relationship with a partner, the more they perceived each other as trustworthy, HIV seronegative, and less likely to transmit HIV. Forgetfulness, time constraints, not wanting to interrupt foreplay, unavailability when needed, and being under the influence of alcohol were additional reasons that men engaged in sex without a condom.

#### Inaccurate information undermines HIV testing

There was broad awareness of the availability of a test to diagnose HIV infection. Despite the availability of this rapid diagnostic technology, HIV testing was not reported as a frequent behavior among the participants. HIV testing was undermined by fatalist views that little can be done to help a person with HIV, either medically or socially, and infection was viewed as a death sentence. Some respondents believed that those who were diagnosed with HIV died sooner than those who remained undiagnosed. Others showed ambivalence towards HIV testing, doubting the validity of the test, and quality of HIV care provided in the health care system. The following respondent believed that a person with an HIV infection would suffer more and potentially die of AIDS sooner if they were informed of the infection than if they remained unaware.

**MSM Respondent:** Well, I strongly agree with what [Participant 2 and 4] have said. As he said, if you are not aware, you can’t be hurt by [HIV] so knowing I may have HIV will put me under a lot of stress and that will probably kill me earlier than it should. So I would not go for the test.

**-** Kumasi, Focus Group 7

This respondent’s misguided understanding of HIV and management options contributed to his avoidance of HIV testing. Furthermore, this sentiment was echoed by several of his peers.

#### Stigma undermines HIV testing

Additionally, fear of knowing their HIV status and the social consequences thereafter were cited as major deterrents to HIV testing. The following respondent explained that because of his timid disposition, he believed that he would be deeply distraught if he found out that he has an HIV infection. He also feared that this would profoundly impact his quality of life and ability to work productively.

**Facilitator:** … do you have plans of testing [for HIV]?

**MSM Respondent:** Not really, I’m a timid person so if I should find out that I am positive, maybe it will affect me very much and maybe it will affect my career even as a whole. So, I really don’t have any plans. And I don’t intend to.

**-** Kumasi, Focus Group 1

Stigma played a central role as a barrier to HIV testing. This stigma was a result of several factors, including being HIV-infected, going to a clinic for HIV testing (regardless of infection status), and identifying as MSM. Participants feared the implications of an HIV infection to their social, family, and professional lives, which was compounded by a perceived lack of confidentiality among health care providers. One respondent narrated how MSM seeking HIV related care in hospitals encounter stigma throughout their time in hospitals, from entrance to exit, regardless of whether they were infected with HIV:

**MSM Respondent:** From the beginning, when you go to the hospital, [when] the doctors find out that you have contracted this virus, [and] after leaving the office, people around will be looking at you even though you may not be infected by the virus. So because of this not all of us have been able to make it [for testing].

**-** Manya Krobo, Focus Group 1

Ultimately, HIV testing among MSM was hindered by fear of knowing HIV status and potential associated stigmas and stressors, in addition to issues of accessibility and utility that were similar to the barriers to condom use.

#### Positive attitudes towards HIV prevention exist among MSM

While negative experiences with HIV prevention resources dominated the group discussions, there was evidence in each community (Table 4) that MSM also had positive and meaningful engagement with HIV prevention. Generally, though not always, there was an understanding of the value of condoms as a form of HIV prevention in addition to being a strategy to prevent pregnancy. MSM who consistently used condoms during sex expressed beliefs in the usefulness of condoms. A personal history of an STI was a motivator to use condoms. Other reasons cited for condom use included: increased pleasure, decreased risk of contracting HIV and other STIs, to protect current or future partners, perceived control over sexual health, and to remain healthy and sustain their longevity into the future. Health education and positive interactions with health care providers who advised participants on how to obtain free or cheap condoms and lubricants contributed to continued condom usage. The potential impact of HIV infection on a career such as playing football was a sufficient reason for consistent condom use, as explained by this respondent:

**MSM Respondent:** I think it’s easy for me [to use condoms] because I don’t want to get any disease from the person with whom I’m having sex with. Because, as I said earlier, I don’t want to get any disease so I wouldn’t have any difficulty in my contract to play football in any country in my career. We footballers do [medical examinations] and through having these condoms, I will be able to reach the place I want to go.

**-** Kumasi, Focus Group 2

Similarly, other men acknowledged that knowing their HIV infection status and getting appropriate and prompt treatment supports their health. They also suggested that if people within their social and sexual networks knew their HIV infection status it would be an important step in keeping each other healthy. Experiencing symptoms of STIs, engagement in self-perceived high-risk sexual behavior, and encouragement from leaders in their peer-network, sexual partners, or media campaigns were identified as persuasive factors for HIV testing.

### Health care climate factors that influence HIV prevention

MSM perceptions of the health care system were affected by the extent to which the overall health care climate respected their right to self-expression. Stigmatizing experiences were cited as reasons that MSM limited the frequency of interactions with health care providers. Additionally, negative experiences of an individual MSM may propagate and influence the health-seeking behaviors of other members of their peer-network.

#### MSM are not free to be themselves

The ability to freely express one’s health concerns to health care providers was an important aspect of the health care experience for MSM; however, MSM did not feel supported to freely express or disclose their sexual identity while seeking care. Even though health clinics are core nodes in the HIV prevention and care infrastructure (e.g., HIV counseling, testing, treatment), they were perceived by MSM as places to be avoided. These avoidances were not direct rejections of HIV prevention services; rather, they were a defense mechanism aimed at self-preservation against negative interactions with health care providers. MSM attitudes towards health care providers were informed by past experiences such as inattentiveness to their needs, expression of personal disapproval of their sexual behaviors, and outright refusal to provide clinical care. Hence, MSM pursued alternate sources for health advice, such as MSM peers and the Internet. Accounts of MSM who had successfully developed supportive relationships with their health care provider were rare in comparison to a generally consistent narrative of distrust in engaging the health care system for HIV prevention services. In the following quote, one participant indicated that he would use the Internet or seek over-the-counter remedies to self-treat symptoms of STIs, rather than risk a potential for a breach of confidentiality and subsequent exposure to stigma by hospital/clinic based care:

**Facilitator:** Who do you see when you have a sexual health related issue?

**MSM Participant**: I think mostly I [Google] to find a solution and if there is no solution, I contact the pharmacy.

**Facilitator:** Why do you choose this option?

**MSM Participant:** I think it is the safest to avoid stigmatization.

**Facilitator:** Why don’t you go to the hospital?

**MSM Participant**: The hospitals in this community have this stigmatization against [MSM] so if they get to know what we do or who we are, it becomes a problem living in the community. So normally to avoid this, we avoid the hospital as much as we can.

-Kumasi, Focus Group 7

The reluctance of MSM to “be themselves” in the presence of health care providers was also influenced by their lack of trust in health care providers’ ability to respect confidentiality or protect their privacy. The perceived consequence of such breaches was that the men’s sexual identities will be exposed and that they will be stigmatized based on their identities and same-gender sexual practices. The concerns about having same-gender practices disclosed ranged from localized breaches within the health clinic (e.g., spreading information among clinic personnel) to more widespread public breaches such as having their “real identity” published in the local newspaper. The concerns regarding privacy also extend into the health care operations and logistics. For example, field-based (mobile) HIV testing is an activity that is frequently done in outdoor settings. According to some participants, the precautions to ensure privacy during HIV testing are insufficient. Consistent with fears about health care provider disclosure of same-gender practices, there was also a lack of confidence that health care providers would protect the confidentiality of HIV test results and a fear that HIV seropositive results would be actively spread to people in their communities.

Health care providers who interact with MSM confirmed that a stigmatizing health care climate negatively affects MSM engagement and retention. In the following conversation, a nurse explained that they are aware that MSM avoided health care settings for fear that a diagnosed HIV infection would be publicly disclosed, in spite of the assurances of health care providers. Furthermore, patients dislike waiting in the clinic because of fear that others in the community will suspect them of having an HIV infection or being MSM, leading to delays in treatment:

**HCP (Public Health Nurse):** … most of them don't come to the health facility when they have problems because they have stigmatization and don't like to come. So most of them would like to be in their houses when they have problems... we've swore that whatever patients do, we do not discuss with others. But they themselves thought that maybe when [they] come for treatment or services, the nurses will disclose. They have fear of disclosure so they don't come.

**Interviewer:** So, again, any other [barriers]?

**HCP:** Yes, and stigma. Maybe this person going is such and such and such, so they don’t come. When [MSM] come, they don’t want to keep long. Maybe thinking that people will come to know. So they don't like to come at all. They will be at their house doing their own thing instead of coming to the clinic for early detection and treatment.

- Accra, Health Care Provider Interview 2

The above explanation from a health care provider regarding the source of MSM’s evasion of clinic-based HIV prevention is consistent with what was described by MSM directly, including fear of discrimination for being MSM and potentially HIV-infected and perceived lack of confidentiality. Consequently, some health care providers conceded that showing greater understanding and kindness towards MSM seeking care is essential. Other health care providers were more laissez-faire, blaming MSM for the health and social consequences of the choices that they have made.

#### Health care providers do not understand the health needs of MSM

MSM believed that health care providers’ lack of understanding of their specific psychosocial issues, vulnerabilities to particular infections, and preventative health needs, limited their ability to provide valuable information to enable them to take control of their own health. For example, MSM stated that health care providers neglected performing genital and anal physical examinations and missed important clinical findings. Furthermore, they promoted abstinence from sex, rather than providing HIV prevention options (e.g. condoms and lubricants), and reprimanded them for engaging in same-gender sexual activities. The MSM respondent in the following quote described being chastised by a nurse who used religion as a tool to dissuade his same-gender sexual activities. This occurred despite the hospital employees’ otherwise friendly demeanor. He recognized this incongruence and suggested that more MSM become trained to be health care providers to enable the MSM community to be more self-sufficient.

**MSM Respondent:** … we already have hospitals where the workers are friendly but when you go there, the nurses will take the Bible and start preaching to you, and say a whole lot of things to you. I think there should be trained providers who are MSM so that, we will take care of ourselves

-Accra, Focus Group 5

However, health care providers generally supported the notion that MSM’s rights need to be respected, which included access to professional health care. Despite providing similar descriptions of these interactions, health care providers and MSM had divergent interpretations of the experiences. Many MSM felt stigmatized when receiving health care advice, and from being in health care facilities even if the health care provider believed that they provided equitable, supportive services. The following two quotes illustrate this discrepancy. In the first, an MSM described an interaction with a physician in which he was given advice that he realized was helpful, but he nonetheless took issue with the physician’s verbal and non-verbal communication. In particular, he described feeling discriminated by the way the doctor and clinical staff looked at him and his friend and by the perceived lack of empathy:

**MSM Respondent:** I went to the hospital… for a check-up and [my friend] was there with me. But the moment I got there and wanted to talk to the doctor, the way the woman watched me and watched my friend, the way the doctor talked about "partner reduction”, “usage of condoms” and “you still don't get it". He's helping me, but the way and the manner that he's talking, he’s got a problem with it.

-Accra, Focus Group 2

In the second exchange, a health care provider discussed his belief that MSM are in need of help because their sexual preferences and activities are “not natural” and contrary to the Bible. The health care provider believed that society is responsible for helping MSM. However, the health care provider also admitted that he is unsure of why men have sex with men – a gap in understanding that patients such as the MSM in the previous quote find problematic when they seek professional health care that is delivered in an insensitive manner.

**HCP (Nurse):** I believe [MSM] are people that really need much attention and care, I don’t know why some of them are into that situation, but I believe the society can help them by sort of education.

**Interviewer:** Why do you think that they need help?

**HCP:** To be honest with you, it is clear that they need help. I understand that is not how it supposed to be, that it’s not natural, and that is not what we are accustomed to. Even the Bible tells us a particular way but they choose to go a particular way so I think they need help. That is what I believe.

-Accra, Health Care Provider Interview 6

Because of these discrepancies in how health care providers and MSM perceive their therapeutic relationship, several health care providers became less capable of providing supportive and inclusive counseling.

#### MSM do not feel that health care providers care about them

MSM felt emotionally disconnected and uncared for by their health care providers. They expressed these feelings as stigma—manifesting through demeaning comments and excessive “lecturing” from health care providers towards MSM because of their engagement in same-gender partnerships and sexual activities, display of mannerisms considered to be effeminate, or perceived HIV seropositive status. For instance, when a participant sought care in the emergency department for a cut resulting from a fight, the attending nurse did not trust his version of the story. Instead, she accused him of being a victim of intimate partner violence and lying about his initial story. The participant was dismayed by the nurse’s unwarranted accusations and verbal abuse, particularly in light of the backstory not necessarily being pertinent to the treatment of his injury.

**MSM Respondent:** I had a fight with someone and the person cut me with a blade. I went to the hospital and the nurse was insulting me, saying ‘Gay, your boyfriend has beat you and you don’t want to tell the truth’. Look at the disgrace.

-Accra, Focus Group 2

Other MSM reported being ridiculed by health care providers who advised them to wear feminine clothes once their sexuality became known. In the following discussion, MSM respondents were asked how welcome they feel within the health care climate of their hospital. The first respondent reported that health care providers who know that a patient engages in same-gender sexual activities may deny treatment or otherwise neglect the patient, which is a consequence of MSM being generally marginalized and discriminated in Ghanaian society. The second respondent affirmed these statements and reiterated sentiments that negative non-verbal cues contribute to the feeling of neglect that MSM experience in health care environments.

**MSM Respondent 1:** I think here in Ghana, we [MSM] are not accepted. So if you should go to doctor and tell him or her your problem and what is happening to you… some of them will not talk to you. They'll tell you to go away.

**MSM Respondent 2:** [Agreeing] [It is] their expression, and their attitude.

-Accra, Focus Group 2

While a majority of the respondents felt that MSM were generally uncared for in the Ghanaian health care system, some reported positive experiences with specific clinics or health care providers, usually using words such as “trust”, “secrets” or “open” to describe their interactions. These MSM felt valued and respected by their health care provider and were more accepting of their health advice on condom usage, HIV testing, and regular medical check-ups. Some health care providers also explained how they initially struggled to connect with a particular MSM patient; however, those whom identified and lessened the gaps between themselves and their patients were able to further their therapeutic relationship and their patients felt freer to divulge clinically relevant details about their relationships and sexual behaviors.

The following two quotes elaborate on how negative health care climates are a barrier to providing high quality care for MSM patients. The health care provider in the first excerpt believed that, in spite of same-gender practices not being accepted in the general Ghanaian society, discriminatory practices were dangerous because they prevented MSM patients from seeking health care.

**HCP:** I would propose MSM should be treated fairly and kindly. It’s an issue that is worrying me personally. It’s true we may not endorse their practice, but they should be treated kindly so that they may come for treatment.

-Manya Krobo, Health Care Provider Interview 5

The health care provider below recognized that, although MSM did receive health care, they generally tried to avoid disclosing their sexual orientation or sexual practices until they required medical attention for a more advanced HIV infection. He further explained that this was because of stigma experienced within their communities, which caused MSM with sexual health concerns to behave differently than patients afflicted by more “socially-acceptable” illnesses, such as malaria. This concerned the health care provider because, while the patient with malaria may be comfortable seeking prompt treatment, an MSM patient with HIV waits until they develop debilitating sequelae of advanced infection.

**HCP (Physician):** They do come for health care all right. They don’t normally come and disclose but one on one they will tell you that this is what has happened - because they need help and before they come, the case would have [become] severe. That is my worry. And I think that because the community or society [does] not accept them, they feel shy coming forward. But when they meet me one on one, they tell me all that has happened. But at the end of the day, you can feel that, they are not comfortable as somebody might come with malaria and expressing it here and there.

-Kumasi, Health Care Provider Interview 10

In both these excerpts, the health care providers advocated for fairness and kindness in the treatment of MSM patients. This also illustrated awareness among health care providers in our sample of issues MSM have raised regarding disconnects in patient-provider relationships.

### Strategies for improving HIV prevention among MSM

In the focus group discussions, participants were asked for their recommendations about how to improve HIV prevention for Ghanaian MSM. Suggestions for improving the HIV prevention for MSM centered on improving and expanding education regarding gender, sexuality and health, as well as instituting legal protections for the human rights of MSM. There is currently a dearth of knowledge surrounding the nature and population size of MSM in Ghana and their particular challenges, which exists at all levels of Ghanaian society, including the general populace, government, health care providers, and MSM themselves.

#### HIV prevention education tailored to MSM enables self-advocacy

Focus group participants suggested that more education about basic HIV prevention should be available to MSM. Potential educational topics include HIV transmission mechanisms, testing and treatment facility locations, and how to stay up to date on the latest research in the field. MSM believed that filling these gaps could help them understand when to seek medical assistance, provide better advice to peers, and ultimately take greater control over their life and well-being. The two MSM respondents below discuss their need for more sexual health knowledge. The first person wishes for increased research directed at investigating issues related to HIV, as well as a mechanism for delivering important findings to the affected populations for them to develop strategies to benefit from the research. Thereafter, a respondent speaks about his desire to know more about antibiotic treatments for STIs so that MSM peer-networks can educate each other and seek over-the-counter treatments from pharmacies, rather than relying on health care providers, particularly those who propagate stigma towards them.

**MSM Respondent 1:** ... We also need more study on this HIV issue because day in and day out there are more research study findings coming…

**MSM Respondent 2:** We are supposed to know much about antibiotic so that when we are in STI trouble, you know that this antibiotic will help me from year to year. So that you can show other friends that, oh, when I'm in need I use this antibiotic. So if you go to the pharmacy or the drug place you can get this antibiotic for your needs.

-Accra, Focus Group 3

Furthermore, knowledge about HIV prevention needs could empower MSM to assemble and engage in self-advocacy. MSM and health care providers both described a need for MSM to be more visible to the general public. With greater public visibility, they could disseminate knowledge to the broader Ghanaian society and the government about the reality of their lives and health needs. However, this kind of large-scale, concerted activism is contingent on the prior self-actualization of participants. MSM wished to instigate a movement towards greater acceptance through disseminating knowledge to others in the community, but they felt that they lack resources and knowledge to do so. In the discussion below, MSM respondents described experiencing frustration that although they were aware of injustices occurring in their communities, they had limited capacity to effect change. The respondent wanted to speak to people in the community to teach them about the lives of MSM. He wanted more resources available to teach MSM about their sexual health needs, instead of staying hidden at home away from public scrutiny – a sentiment that the rest of his group echoed.

**MSM Respondent:** …one of our needs is we want to go out and talk to people. Because we know there's a lot of stuff happening. I can just stay in my house and feel good, but I know my friend is suffering outside, so we go outside and talk to people… About condom usage, STIs.

**Facilitator:** So you want more resources to educate people about STIs?

**MSM Respondent:** Yes, exactly [agreement from group]

-Accra, Focus Group 2

There are many misconceptions and stereotypes about MSM held in Ghanaian society and, to an extent, internalized by MSM themselves. MSM believed that once they can discuss pertinent issues more openly and with greater insight, they would develop the capacity to challenge these assumptions and misconceptions. Below, a respondent described going for an HIV test to see if a rumor he heard about all gay men having HIV was true. He explains that after finding out that he does not have an HIV infection and gaining this piece of knowledge about HIV in gay men, he became capable of challenging other people’s assumptions.

**MSM Respondent:** … I tested about 6 months ago. I heard all gay people have HIV and it made me wonder a lot so I decided to go for the test and it came out negative. So, when I hear someone saying something like that, I can defend it by saying not all gay men have HIV. We can’t challenge them on those assumptions because we haven’t gotten that opportunity…

-Kumasi, Focus Group 5

MSM believed that educational interventions should be delivered in a manner that is empathetic towards their needs and the unique challenges that they face in their day-to-day lives. As described earlier, patient education that is delivered in an uncaring manner by health care providers tended to be poorly received in comparison to approaches to education that involved acceptance of MSM’s life choices and recognition that they are an equal part of Ghanaian society.

#### Education for the public and health care providers to protect MSM human rights

MSM believed that Ghanaian society was intolerant towards same-gender sexual activity. They endured derogatory remarks from people within their communities, were often excluded from social circles, struggled to make friends, and experienced rejection and anger from family. Men who outwardly exhibited more “feminine” appearances and behaviors faced additional insults, violence, and isolation. Many MSM feared the consequences of others knowing about their private lives. As a result, they experienced psychological distress and took measures to conceal their sexual identity from the public, including minimizing contact with the health care system.

Interviews with health care providers supported the finding that prevailing attitudes in Ghana are not supportive of same-gender sexual relationships. There was a shared sense from both MSM and health care providers that the unclear legal status of homosexuality and societal attitudes perpetuate discrimination of MSM. The health care provider quoted below explains how Ghana’s social atmosphere of discrimination towards same-gender relationships and practices take precedent over any legal prohibitions or allowances. Ultimately, the health care provider believes that for public perceptions to change, officials within the judicial system or government need to initiate a social movement towards greater acceptance.

**HCP (Physician assistant):** … our culture does not accept homosexuality and lesbianism. … education [should go] around that we are now in a free society and people are free to do whatever they want, so long as it does not go against the law. Because culture prohibits it – but the law in Ghana does not prohibit it. I don't know if there's anything there in the law books that prohibits lesbianism or homosexuality, you see? But the culture overrides the law, in our eyes, you see? So until the law enforcement or the government comes out in the open to make it clear that all people are free, the MSM will continue to be discriminated against.

-Accra, Health Care Provider Interview 3

Additionally, some focus groups revealed that MSM harbored internalized homophobia from society, expressing negativity towards other MSM with more feminine tendencies, or seeing their own sexuality as an immoral habit from which they needed to refrain. Below, two MSM respondents discussed how people displaying characteristics considered effeminate bring problems upon themselves. The first respondent suggests that men adopt classically “masculine” mannerisms and appearances to avoid the stigmatizing culture, generally pervasive throughout Africa. The second person believed that although violence against males who display gender non-conforming characteristics is not right, the male doing so is essentially asking for trouble.

**MSM Respondent 1:** I think in Ghana, or in Africa this is frowned upon so if we could dress as normal people, it will help. Because I know people who put on eye shadows, mascara, dress up with acrylic paint, handbags and they walk around with everything about them but voices like men.

**MSM Respondent 2:** To add to that, I have been telling people that, to get others to sleep with you isn’t about your physical touch ups… So I guess that's one point of education. Sometimes we bring violence unto ourselves. This stuff is frowned upon so people are ready to get into vigilante justice as practiced in some other countries. And so when they see you in such ornaments, they just pounce at you. They are not justified though, but you are calling it on yourself.

-Accra, Focus Group 5

Agreeing that MSM are marginalized in Ghana, health care providers put forth two options for education and awareness targeted to the general population as potential solutions: Enhancing understanding and compassion towards MSM, or increasing stigmatizing attitudes towards same-gender practices to dissuade people from the “unnatural” habit. MSM in the focus groups, as well as several health care providers, more commonly sided with the former suggestion. They stated that if people in society, including government and health care providers, better understand the reality of same-gender practices it can reduce abuse, liberalize legal frameworks, and improve the patient-centeredness of the overall health care climate. Below, an MSM respondent described the need for society to understand that MSM are like all other people and therefore entitled to the same rights and freedoms. He also stated that there are not many other people who see MSM in this way and that it is quite difficult to live in isolation so long as others are not accepting of their lifestyle.

**MSM Respondent:** …we are also human beings and we will also like to have freedom like others, but those [other people] who think so are very few. And so, if we go by them, we will just feel isolated in the midst of so many [MSM] in the society. I think those who know we are in this community should let [us] be like this.

-Kumasi, Focus Group 5

Contrasting this view, health care providers who voiced opposition towards same-gender practices sometimes believed that MSM are responsible for propagating HIV and, consequently, homosexuality should be reduced and dissuaded in society. The health care provider quoted below believed that leaders in society should promote stringent restrictions to reduce same-gender practices. He believed that there are no effective ways to mitigate the risk of disease transmission among MSM and that they will cause HIV transmission to continue endlessly.

**HCP:** … the politicians have to come in. Our traditional leaders also have to come in. If we institute very formidable rules, it can help us to curb or mitigate that particular act from this society. But because there are no proper measures against that particular act, they are doing it. And it will go on and on and on till it becomes something that cannot be controlled anymore because our leaders have left such issues aside.

-Kumasi, Health Care Provider Interview 3

Suggested solutions to improve the perception of MSM in the public eye focused on media campaigns and government involvement. The Ghana AIDS Commission was cited as a key potential player that could increase awareness of MSM health issues. Proponents of the top-down approach suggested that government addresses stigma towards MSM by supporting their right to free expression in order to begin shifting cultural attitudes. In the quote below, one MSM respondent suggested that since most MSM do not know how to find supportive professional health services, HIV prevention interventions should take place in social venues where MSM tend to congregate.

**MSM Respondent:** [Improving our lives] is about educating us on our social grounds. There are most of us who don’t even know where to go for help when they need one. So if there is sometime like bars and clubs where normally hangout, if people can be educated there, it’s the only simple way they can get the message through.

-Accra, Focus Group 5

Focus group participants and healthcare providers both commonly recommended more MSM-specific health care facilities and health care providers who self-identify as MSM as strategies to increase knowledge and sensitivity towards MSM health needs. Supporters saw these as logical strategies to resolve issues experienced with health care providers who have limited experience working with MSM. Those opposed to MSM-specific health facilities feared that it would create greater social segregation and impede the normalization of same-gender relationships in Ghanaian society. Beyond greater provision of HIV prevention tools, MSM believed that sustainable strategies to improve their lives should enhance understanding of their own sexuality. This is important at the level of MSM themselves to reduce internalized stigma, and enable better self-care, advocacy, and assembly with other MSM. It is also important in society at-large to reduce stigma, which mars the quality of life of the LGBT community and hinders more socially just policy development.

## Discussion

Ghanaian MSM face a multitude of obstacles that systematically impede successful HIV prevention strategies. Through focus group discussions, we sought to understand the experiences of MSM using HIV prevention resources, what factors influenced their utilization of prevention resources, and their self-identified strategies to improve sustainable HIV and STI prevention in Ghanaian communities. There were predominantly negative perceptions towards condoms and HIV testing, grounded in dissatisfaction with the quality of condoms and misconceptions towards the utility and effectiveness of both condoms and HIV testing. We found counter-narratives describing positive outlooks towards condoms and HIV testing, usually because participants believed that they were tools to sustain a healthier, more productive life. However, negative perceptions were compounded because MSM view the health care system, which normally should be an important vehicle for preventative health education [18], as being unsupportive of their basic human rights to respect, equitable treatment, and free-expression. Finally, suggestions for effective strategies to improve HIV prevention for MSM in Ghana reflected a desire for a multi-level approach to educating MSM, the general population, and health care providers on HIV and human rights.

In Ghana, the issues above are presented in the context a nebulous legal framework around the status of MSM that is used as a justification for their persecution and is reflective of homophobic attitudes in the general population [15–17]. The current HIV prevalence within the MSM subpopulation is nearly fifteen times higher than the general population, a consequence of relatively low uptake and utilization of HIV prevention strategies [24]. Perceived lack of self-determination in the health care system [29], as well as felt stigma [30] decrease initial access to care and follow-up with a health care provider in other HIV-infected populations. These factors contribute to a treacherous cycle in which MSM are more susceptible to HIV infection and take longer to receive a diagnosis and proper advice to stay healthy and prevent further transmission. Further intersectional stigmas from HIV infection, sex work, and mental illness may compound the sexuality-associated stigma faced by MSM, further impairing their ability to live freely within Ghanaian communities and institutions [31].

Sustainable HIV prevention strategies must realize the importance of improving the perceptions of MSM towards the health care system and existing, efficacious prevention strategies. Currently, MSM are distrustful of the health care system, its practitioners, and the products that it prescribes. There are many misconceptions surrounding key HIV prevention strategies including the beliefs that condoms are ineffective for HIV/STI prevention, HIV testing is inaccurate, nothing can be done to help someone with HIV, and knowing one’s infection status will accelerate their clinical decline and death from AIDS. These ideas persist even among focus group participants living in Accra and Kumasi – larger urban centers in which MSM have been found to have higher access to HIV prevention services than the national average. [4] Additionally, participants frequently saw condom usage as a sign of distrust in the partner and believed that they would be unnecessary in a trusting relationship, seeing HIV and STIs as things that could intentionally and maliciously be passed on from a partner. There was a broad spectrum of how much weight people placed on STI prevention, compared to contraception or having a more pleasurable sexual experience when deciding to use condoms. These findings are consistent with previous data describing low knowledge about the transmission of HIV and other STIs among Ghanaian MSM [6].

Nonetheless, MSM still find subversive ways to exercise autonomy in light of an unsupportive health care environment. For example, MSM will seek health care information and resources from alternative sources, such as pharmacies, peers, and the Internet, reflecting their underlying desire to live a healthy life. However, these sources may not provide trustworthy, evidence-based information and encourage ill-informed decisions. These actions are borne from fear of abuse from health care providers and show that MSM pursue alternative routes for self-care when the health care climate is not perceived as “safe”. In spite of their limitations, these alternative sources could be leveraged by health care providers or the government to disseminate higher quality information to those who access them.

Self-determination theory (SDT) provides a plausible framework to explore and understand health care provider-MSM relationships in the Ghanaian context. SDT posits that when patients experience greater autonomy support from their health care providers, they are more internally motivated to take actions to accomplish their health promotion and treatment goals [32]. According to SDT, the three components needed to optimize health behaviors are autonomy, competence, and relatedness. Autonomy support refers to aspects of the health care system that make patients feel comfortable describing their medical and social background, as well as enabling them to take control of their own health decisions. Competence support helps empower the patient to attain their goals regarding health and well-being through providing them with necessary skills and resources. Relatedness support reflects kindness and empathy towards the individual. The different elements of SDT play vital roles at each step along the HIV care continuum, from initial connection with health care providers, to HIV testing, to short- and long-term retention [29].

When MSM feel that health care providers do not provide them with autonomy over their health, either because of overly authoritative lecturing, poor assurance of confidentiality, or intimidating or fear-inducing interactions, they feel less inclined to disclose potentially relevant health information. Health care providers who give rigid advice, particularly when it is counter to the normal habits or values of the patient, can diminish the patient’s sense of control over their options of how to lead their life. Notably, Ghanaian MSM who reported greater experiences of autonomy support from their healthcare provider were more likely to report condom use for anal, oral, and vaginal sex [24]

Competence support lacks because MSM do not feel that health care providers understand their unique needs, diminishing their ability to equip patients with the skills and knowledge needed to remain healthy. These knowledge gaps take shape primarily in two ways: 1) Health care providers having limited understanding of diseases and disease processes more prevalent among MSM (e.g. HIV, common STIs, psychosocial issues), and specific prevention strategies, and 2) Health care providers failing to counsel in a patient-centered manner due to limited understanding of individuals’ motivations for same-gender activities and their related challenges. Rather, MSM feel that they are judged and given advice from health care providers that reflects a moralistic evaluation of health and risk behavior.

Multiple suggestions are given to help remedy this situation. On a large scale, awareness campaigns or greater support from the Ghanaian government can focus attention on the importance of understanding the needs of MSM patients. These campaigns could promote awareness of MSM-specific needs for increased sexual health counseling (including various options other than abstinence) and more targeted screening questions and examinations for STIs, HIV, and psychosocial concerns. This could be implemented through more socially accepted entry points, such as within broader discussions of HIV prevention or gender based violence. Additionally, focus group participants expressed that they would like to interact with health care providers who are also MSM, or that there should be clinics that specialize in providing care to MSM. Whether such a clinic could exist in the current social climate remains to be seen. Creating such an institution could risk further segregating MSM from society, and the health care providers and MSM patients involved may also be at risk of legal and physical threats. A focus on making existing clinics and hospitals more patient-centered may be a feasible approach to achieving the aims. This could include utilizing cognitive-behavioral techniques in HIV prevention counseling [33], or a variety of changes in clinic operations from increasing privacy for HIV testing, to using health promotion materials that are more applicable to MSM (e.g. pro-condom advertisements that focus on anal intercourse rather than contraception).

Violations of relatedness support were manifest in both overt and covert aggressions against MSM patients. These experiences range from outbursts of anger and derogatory comments towards patients based on their sexual histories, to health care providers falsely ascribing illnesses to patient sexuality, to a general unease experienced by some MSM during their interactions with health care providers. The highly contentious and misunderstood nature of same-gender relationships in general society is central in each of these aggressions, whether subtle or bold. Ultimately, breaches of the three core tenants of SDT result in a health care system that does not provide sufficient care or respect MSM human rights and dissuades engagement.

Educating the general population about the role of MSM in society and their specific health needs is at the crux of building mutual trust between MSM and health care providers. There still remain vital questions surrounding exactly what education needs to be provided, who is responsible or best positioned to provide such education, and how knowledge should be disseminated. Ultimately, Ghanaian MSM should have a central role in producing and disseminating knowledge to ensure that educational interventions contain accurate content and are delivered in culturally appropriate manners.

MSM in Ghana are a diverse population with respect to age, geography, ethnicity, religion, and socioeconomic status, but using a combination of strategies may develop their capacities to teach each other and the rest of society. These strategies to encourage HIV prevention could include peer-education networks and motivational interviewing, which have been shown to improve uptake of prevention resources such as HIV testing among MSM in other settings [34, 35]. Focus groups reveal that MSM in a given peer-network often have similar perspectives towards issues and have similar health-seeking behaviors (e.g. going to pharmacies for advice instead of a clinic) and that individuals find the group to be a valuable life-resource. These peer-networks can be utilized to build more formal, well-informed peer-education programs.

Direct knowledge exchange between MSM and health care providers may also be vital. Health care providers have unique relationships with patients bound by confidentiality and a responsibility to promote their well-being, as well as a privileged role as educators and advocates in society. Health care providers who are educated about MSM and understand their health needs may have the necessary lobbying power to affect a paradigm shift in the thinking of broader society. In addition, the media has had a large role in changing public perceptions and increasing awareness about public health interventions, as has been seen with HIV testing. Participants felt that greater media and government involvement could have an important role in normalizing same-gender relationships and making Ghanaian society more understanding of the realities of MSM.

There were several examples of health care providers who support the self-determination of MSM by advocating for patient-centered approaches to working with MSM and believing that recognizing them equal citizens is important to sustainable HIV prevention. There were also examples in each of the cities of focus groups with MSM who had positive experiences interacting with health care providers, seeing them as empathetic, trustworthy, and competent in providing care. Although these positive interactions were in the minority and likely overrepresented since all interviewed health care providers had a history of working with MSM patients, they provide tangible examples of how health care could be more patient-centered within the Ghanaian cultural context. MSM looked positively upon health care providers who treated them with kindness and maintained confidentiality. When a health care provider supplied free or discounted condoms, went out of their way to prevent discrimination in clinics, took extra time after work to organize support groups, or listened to the struggles their patients had with families and friends, MSM felt engaged with their provider and believed that the health care system is working in their best interests. There is room for health care providers to learn from MSM, as well as from their colleagues. Given the existing hazards of being MSM and of openly working with MSM it is vital to develop safe venues for role models to advocate and educate others. These hopeful examples demonstrate that an improved approach to HIV prevention for MSM is attainable within an unsupportive sociocultural context in Ghana, and beyond.

There are several limitations to our findings, which must be considered as further steps to advance HIV prevention in Ghana are taken. The MSM focus groups and health care provider interviews have provided us with perspectives that, while robust, may not be reflective of a broader network of MSM and health care providers in Ghana. For example, although we made a strong attempt to include views from different geographical, social, and cultural strata by interviewing people in the Accra, Kumasi, and Manya Krobo metropolitan areas, there were many areas of Ghana where we were not able to collect data. Our study also consisted primarily of young MSM (<25 years old) whose views may differ from those expressed by older MSM. While the experiences of MSM and health care providers in Ghana are potentially similar to those of people in other countries, particularly in West Africa, these reflections were made under social and political circumstances that are unique to Ghana. Since this study’s focus groups were comprised of peer-networks of MSM, the views expressed here may differ from more socially isolated MSM who do not belong to similar groups.

Reducing the number of new HIV infections is a critical goal of international development identified in the United Nations’ Millennium Development Goals and reiterated in their more recent Sustainable Development Goals [1, 36]. This study forms the basis for future work to develop sustainable interventions to improve HIV prevention in Ghana among MSM. Education strategies must be poised to engage people at multiple levels and may utilize a combination of traditional non-formal mediums (e.g. social activism, information exchange in social networks) and formal mediums (e.g. print media, school lessons, television campaigns), as well as novel technologies (e.g. mobile technologies, social media, E-learning). Additionally, in light of negative views towards currently available prevention methods (e.g. condoms, abstinence), the acceptability of additional prevention options such as rectal microbicides, pre-exposure prophylaxis, and post-exposure prophylaxis should be investigated. Further research could focus on evidence-based education and support programs, the structure and function of MSM peer-networks, perspectives of other MSM subpopulations (e.g. older age, rural, gay-identifying vs. non-gay-identifying), and alternate sources of care that people utilize when avoiding the health care system.

The challenges for MSM in Ghana are plentiful, but the movement towards greater social equity is building momentum and progressive changes seen around the world may provide guidance for a positive paradigm shift. Strengthening the capacity for self-determination and equity among MSM within society and the health care system will provide a foundation for improving their well-being – in regards to HIV prevention, as well as all other areas of health.

## Conclusions

MSM in Ghana are exposed to negative health care climates, where condom utilization, HIV testing rates, and sexual health education for MSM are undermined by a generally unsupportive cultural and social context. At the core of this issue, strides must be made to improve the understanding of all people in society about the realities of same-gender relationships and the specific health needs of MSM. These findings contribute to community stakeholder (e.g. MSM, health care providers, traditional leaders, government policy makers) knowledge to inform development of HIV prevention interventions for MSM in Ghana, such as culturally appropriate sexual health education, peer-counseling programs, continuing medical education for health care providers, and novel digital technologies to connect individuals with resources supportive of MSM.

## Abbreviations

AIDS: Acquired immune deficiency syndrome
CPEHRG: Center for Popular Education and Human Rights Ghana
GALAG: Gay and Lesbian Association of Ghana
HCP: Health care provider
HIV: Human immunodeficiency virus
KAPPA: Kumasi & Accra Project to Prevent AIDS
LGBT: Lesbian, Gay, Bisexual, and Transgender
MSM: Men who have Sex with Men
NGO: Non-Governmental Organization
PORSH: Priorities and Rights in Sexual Health
SDT: Self-determination theory
STD: Sexually transmitted disease
STI: Sexually transmitted infection
